# CRISPR/Cas9 Genome Editing in the Diamondback Moth: Current Progress, Challenges, and Prospects

**DOI:** 10.3390/ijms26041515

**Published:** 2025-02-11

**Authors:** Muhammad Asad, Yanpeng Chang, Jianying Liao, Guang Yang

**Affiliations:** 1 State Key Laboratory of Agricultural and Forestry Biosecurity, Institute of Applied Ecology, Fujian Agriculture and Forestry University, Fuzhou 350002, China; axadch@fafu.edu.cn (M.A.); fsrmchang@163.com (Y.C.); jianyingliao0317@126.com (J.L.); 2 Joint International Research Laboratory of Ecological Pest Control, Ministry of Education, Fuzhou 350002, China; 3 Ministerial and Provincial Joint Innovation Centre for Safety Production of Cross-Strait Crops, Fujian Agriculture and Forestry University, Fuzhou 350002, China; 4 Key Laboratory of Integrated Pest Management for Fujian-Taiwan Crops, Ministry of Agriculture, Fuzhou 350002, China; 5 Key Laboratory of Green Pest Control, Fujian Province University, Fuzhou 350002, China

**Keywords:** site-directed mutagenesis, genetic control, efficiency of CRISPR/Cas9 system, *Plutella xylostella*, gene drive, genetic modification

## Abstract

The development of site-specific genome-editing tools like CRISPR (clustered regularly interspaced short palindromic repeat) and its associated protein, Cas9, is revolutionizing genetic engineering with its highly efficient mechanism, offering the potential for effective pest management. Recently, CRISPR/Cas9 gene-editing has been extensively utilized in the management of the diamondback moth, *Plutella xylostella* (L.), a highly destructive pest of vegetable crops, for different purposes, such as gene function analysis and genetic control. However, the progress related to this gene-editing tool in *P. xylostella* has not yet been summarized. This review highlights the progress and applications of CRISPR/Cas9 in uncovering the genes critical for development, reproduction, and insecticide resistance in *P. xylostella*. Moreover, the progress related to the CRISPR/Cas9 gene drive for population suppression and modifications has also been discussed. In addition to the significant progress made, challenges such as low germline editing efficiency and limited homology-directed repair remain obstacles to its widespread application. To address these limitations, we have discussed the different strategies that are anticipated to improve the efficiency of CRISPR/Cas9, paving the way to it becoming a pivotal tool in sustainable pest management. Therefore, the present review will help researchers in the future enhance the efficiency of the CRISPR/Cas9 system and use it to manage the diamondback moth.

## 1. Introduction

**Figure 1:**
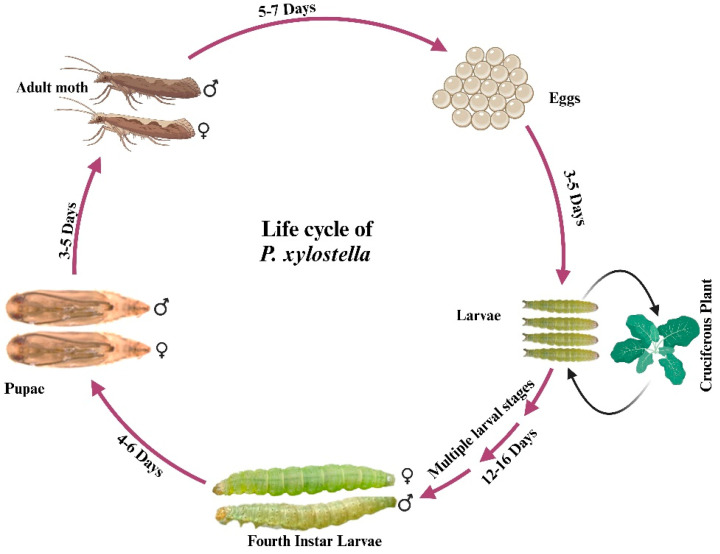
Life cycle of *P. xylostella*. The life cycle of *P. xylostella* (from hatching to adulthood) typically lasts 18–25 days, varying based on temperature and geographic location. The larval stage (from the first to the fourth instar) is the longest phase of the diamondback moth’s life cycle.

The diamondback moth (DBM), *Plutella xylostella* (L.) (Lepidoptera: Plutellidae), is a widespread pest species within the Lepidopteran group that causes significant damage to cruciferous crops worldwide [1]. *P. xylostella* is known as an oligophagous pest as it feeds on both wild and cultivated cruciferous vegetables (Cruciferae), including some commercially and economically significant vegetables like cauliflower, cabbage, and canola [2]. The damage caused by *P. xylostella*, along with management costs, results in estimated global economic losses of USD 4–5 billion annually [1]. The life cycle of the *P. xylostella* varies with temperature, ranging from 4 to 20 generations per year [1,2,3]. First-instar larvae act as leaf miners, feeding on the spongy tissue of leaves. As they progress to later instars (second, third, and fourth), the larvae transition to feeding on the leaf surface, consuming the outer green layer, buds, flowers, stems, and seeds (Figure 1). This feeding behavior leads to significant crop damage, reducing both yield and quality of crops [1,2]. Insecticides have traditionally been the main method for controlling *P. xylostella* populations. However, the moth has evolved resistance to almost all major groups of insecticides, making it challenging to manage in agricultural settings [1,4,5]. Given these challenges, there is a critical need for innovative and effective strategies, such as genetic-based control methods [6]. These include RNA interference (RNAi) for gene silencing, transgenic approaches like crops expressing *Bacillus thuringiensis* (*Bt*) toxins, and clustered regularly interspaced short palindromic repeat and its associated protein Cas9 (CRISPR/Cas9) for precise genome editing, all of which offer sustainable alternatives to chemical pesticides [6]. Among these strategies, the CRISPR/Cas9 system is known to be a promising tool to develop advanced pest control strategies, including gene drive systems for population suppression [7,8] and genetic sterile insect techniques (gSIT) such as the precision-guided sterile insect technique (pgSIT) [9,10].

**Figure 2:**
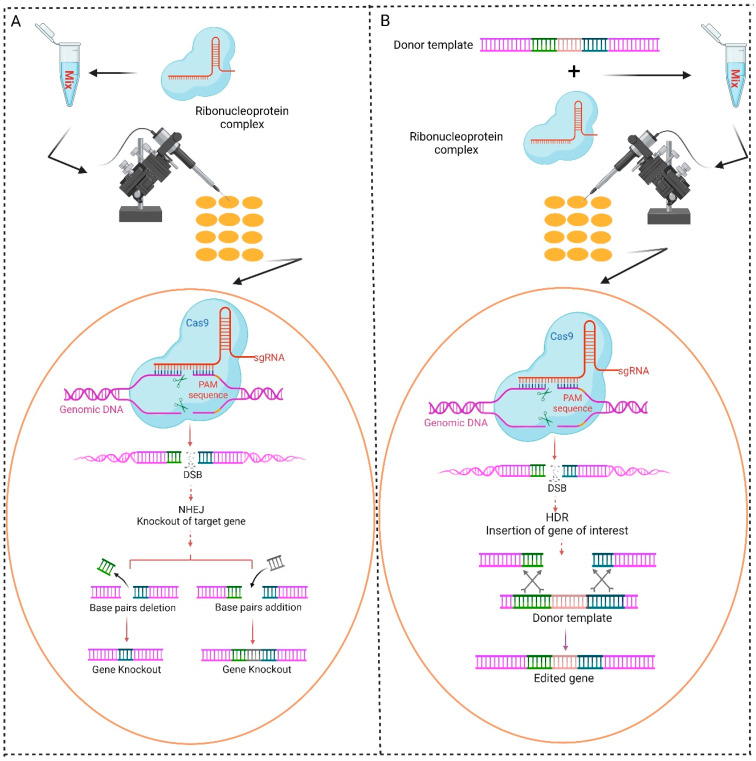
Schematic illustration of the CRISPR/Cas9 system in the diamondback moth. Two basic methods of the CRISPR/Cas9 genome-editing system are being used in the diamondback moth. (**A**) The knockout method: a mixture of ribonucleoprotein (gRNA + Cas9) is injected into the eggs of the diamondback moth. The gRNA binds to a specific target gene, and Cas9 creates a DSB. The cleaved DNA repairs through NHEJ and directs the knockout of the particular target gene. (**B**) The knock-in method: a mixture of ribonucleoprotein and donor plasmid is injected into the eggs of the diamondback moth. The gRNA binds to the target gene and Cas9 creates a DSB. The cleaved DNA repairs through HDR and copies the donor DNA fragment to the cleaved target site.

As early as 1987, the structure of CRISPR was first identified in bacterial species known as *Escherichia coli* [11]. However, the same CRISPR structures were further discovered in other various bacterial species, and the abbreviation of this system was developed in 2002 [12]. CRISPR gene loci are mainly located adjacent to Cas9 genes and typically comprise multiple noncontiguous direct repeats that have been divided by flexible sequences referred to spacers [13]. Since 2013, the CRISPR/Cas system has been commonly employed in different types of biological research, and CRISPR/Cas9 is the most utilized variant of this system [14,15]. The CRISPR/Cas9 gene-editing system has dramatically improved the field of genetic engineering due to its accurate and highly efficient mechanism [16]. The guide RNA (gRNA) is an artificial synthesized RNA molecule that is complementary to the target sequence in the genome of an organism and is essential for the proper functioning of this system. Cas9 proteins are known as molecular scissors, which are paired with the gRNA. The Cas9–gRNA complex initiates the precise double-stranded break (DSB) in the target DNA sequence after locating it in the genome (Figure 2). After the DSB, the biological mechanisms of DNA repairing initiate, including non-homologous end joining (NHEJ) and homology-directed repair (HDR) [17,18,19]. NHEJ frequently triggers the minor deletions or insertions known as indels, which eventually disrupt the open-reading frame and result in gene knockout [18]. On the contrary, HDR can be used to create targeted genetic alterations by introducing a DNA template alongside the CRISPR/Cas9 components, facilitating accurate gene editing with the desired alterations [18,20,21,22]. Due to its efficiency and specificity, the CRISPR/Cas9 system has been effectively used in various organisms, including plants [23], microbes [24], animals [25], insects [26,27], and humans [28].

Moreover, this system presents an optimistic method for pest control within the field of pest management. This technique involves targeting certain genes that are crucial for the survival or reproduction of pests and that may be used to develop transgenic crops, which are resistant to pest attack, by altering the genes associated with susceptibility to pests, thereby decreasing the need for chemical pesticides and encouraging sustainable agriculture practices. In addition, the use of the CRISPR/Cas9 system has been employed to investigate the drug resistance mechanisms in pathogens, demonstrating its promise in tackling issues linked to pest and pathogen control. CRISPR/Cas9 technology shows several benefits, such as its adaptability and effectiveness in facilitating gene editing in different organisms [20]. Moreover, it has been demonstrated that ribonucleoprotein complexes and DNA-free genome editing reduce off-target effects, ultimately improving the accuracy of gene editing through this technology [29]. In comparison to prior gene-editing technologies, CRISPR/Cas9 exhibits a notable degree of efficiency and cost-effectiveness, thereby expediting advancements in the fields of research and development.

## 2. Applications of CRISPR/Cas9-Based Gene Knockout System in Diamondback Moth

The emergence of CRISPR/Cas9-based gene editing has shown great potential in pest management applications. Before its emergence, research on the gene function in the *P. xylostella* was limited due to the inefficacy of RNAi and the absence of direct genome-editing technology. In 2016, the effective use of CRISPR/Cas9 to specifically target *Abdominal A* gene (*Pxabd*-*A*) in *P. xylostella* validated the promise of utilizing this approach to disturb the genes in agricultural pests [30]. Subsequently, by using this system in *P. xylostella*, the understanding of genes associated with growth, development, and reproduction gradually improved, leading to significant advancements in pesticide and *Bt* resistance mechanisms (Table 1).

### 2.1. Development and Reproduction

CRISPR/Cas9 technology has significant potential as a tool for gene function study, which has effectively been used to uncover the roles of genes related to development and reproduction in the diamondback moth. *Vitellogenin receptor* (*VGR*) and *Vitellogenin* (*Vg*) genes play crucial functions in insect oocyte maturation and embryonic development. Disruption of *PxVg* and *PxVgR* through the CRISPR/Cas9 system leads to a reduction in ovary size and decreased hatching rates, while egg production remains unaffected [32,33]. *Serine protease* 2 (*Ser2*) belongs to the Serine proteases gene family and serves as a crucial constituent of insect seminal fluid proteins. A mutation in *PxSer2* leads to a dominant form of male infertility that is inherited by the next generation [34]. Vitelline membrane proteins (VMPs) are key components involved in the formation of the insect vitelline membrane layer, which is essential for oogenesis and embryonic development. Knockout of *PxVMP26* leads to abnormal eggs with a lower hatching rate, while egg production remains unaffected [35]. Piwi protein plays a pivotal role in animal germ cell development and transposon silencing through its interaction with piRNA-associated small non-coding RNAs. Knockout of *PxPiwi* gene prolongs the pupal stage, leading to abnormal eclosion [36]. Deletion of biogenic amine *tyramine receptor 1* (*PxTAR1*) inhibits sex pheromone biosynthesis and ovarian development, resulting in reduced mature oocytes and decreased egg production [37]. *Ovary-serine protease* (*Osp*) is crucial for the process of oogenesis and the development of ovaries. The absence of *PxOsp* results in recessive infertility in females, whereas male fertility stays unaffected [38].

Utilizing CRISPR/Cas9 in the *P. xylostella* not only reveals the conservation of genes typically involved in reproductive development but also sheds light on the participation of non-traditional reproductive development genes. For example, the transcription factors (TFs) may have significant functions in growth and development, sexual dimorphism, and pesticide resistance. Disruption of the *Homeobox* transcription factor gene cluster leads to a reduction in the egg hatching rate [31]. Dicer, a member of the RNase III family endonucleases, shows precise recognition and cleavage of dsRNA. Knockout mutants of *PxDcr-1* display higher mortality rates, lower eclosion rates, reduced egg production, decreased hatching rates, and ovarian abnormalities, whereas no significant differences are seen between *PxDcr-2* knockout mutants and wild-type individuals in terms of their developmental and reproductive characteristics [39].

### 2.2. Pigmentation

The study of phenotypic marker genes is essential to screening transgenic insects for the development of specific genetic control systems. When modeling gene drives and assessing their effectiveness on target insects, it is crucial to initially target endogenous phenotypic genes. Knockout of *PxYellow* causes modified body pigmentation across larvae, pupae, and adults but does not cause significant differences in adult oviposition or egg hatching rate. This suggests that *PxYellow* does not have direct role in growth, development, and reproductive regulation, indicating its potential for use as a genetic marker for development of genetic control technologies [42]. The eye color gene has been served as an obvious marker in many prototype strategies for genetic control of insects. CRISPR/Cas9-based disruption of *kynurenine 3-hydroxylase* and *Cardinal* in the diamondback moth results to yellow compound eyes in adults, along with changes in brain, ovary, and testis pigmentation [40]. *Ebony* gene serves as a core enzyme involved in dopamine conversion to N-β-alanyldopamine and plays an important function in the melanin production. Disruption of *PxEbony* results in the development of dark pigmentation in the bodies of larvae, pupae, and adult insects. Additionally, there is a notable decrease in the rates of egg hatching and the survival of larvae when compared to the wild-type strain [41].

### 2.3. Sex Determination

Insects have developed intricate and complex mechanisms for sex determination, showing significant variations. Presently, the procedures for determining sex have been thoroughly studied in Dipteran insects such as *Drosophila*. Nevertheless, there is a lack of research on the processes of sex determination in Lepidopteran insects. However, the use of the CRISPR/Cas9 system has allowed for the study of the partial sex determination mechanism in the lepidopteran model insect *B. mori*. For *P. xylostella*, only a few genes related to sex determination pathways have been studies by utilizing the CRISPR/Cas9 genome-editing tool. *Doublesex* (*Dsx*) is a pivotal gene in insects that is essential for sex determination. It plays a critical part in the process of sexual differentiation and development. In the diamondback moth, the *Dsx* gene possesses a single transcript for males and three transcripts for females. The CRISPR/Cas9 method has been utilized to deliberately introduce mutations in both the male- and female-specific isoforms, as well as independently targeting each particular isoform for males and females. The male mutants exhibits the expression of female-specific transcripts, while the female mutants showed the expression of male-specific transcripts, ultimately leading to gender-specific sterility [43]. The P-element somatic inhibitor (PSI) is crucial for promoting male-specific splicing of the *Dsx* gene, which is essential for male sexual differentiation. Knockout of *PxPSI* results in the malformation of male reproductive organs, leading to infertility [44]. Seminal fluid proteins (SFPs) are major determinants of reproductive success between sexes. Knockout of one SFPs gene(*PxSast1*) reduces male mating ability and fertilization capacity by suppressing the expression of female follicular genes, leading to defects in oogenesis [45].

### 2.4. Circadian Rhythms

The biological clock regulates the rhythmicity of metabolism, physiology, and behavior in organisms. The majority of organisms possess a circadian rhythm that is regulated by a molecular oscillator consisting of multiple clock genes that are constant across species. These clock genes include timeless (tim), period (per), cryptochrome (cry), clock (clk), and cycle (cyc) [71,72]. The absence of *PxCry1* disrupts rhythmic locomotion under light–dark conditions and eliminates rhythmicity under continuous darkness. This highlights that *PxCry1* is crucial for the regulation of diurnal rhythm in the diamondback moth [46]. Deletion of *PxCry2* or *Pxper* eliminates the peak activity after the absence of light during light–dark cycles. Furthermore, the lack of *PxCry2* diminishes overall activity, which is essential for sustaining internal rhythms in a state of continuous darkness [48]. Phototaxis, the movement in response to light, is an important ecological trait for nocturnal insects. Three opsin genes, namely, LW-opsin, BL-opsin, and UV-opsin, have been identified in the genome of the diamondback moth. These genes are mainly expressed in the head region, with *LW-opsin* having the highest expression level. Knockout mutants lacking *PxLW*-*opsin* show reduced phototaxis towards various white light wavelengths and impaired perception of white light, green light, and infrared light [47].

### 2.5. Ecological Adaptability

Insects play a critical role in ecosystems by occupying essential ecological niches, largely due to their impressive capacity for adaptation. This adaptability is evident in their ability to thrive in various environments through host adaptation, which involves adjustments in diet, reproductive strategies, and behavior. A key impetus for the evolutionary adaptation of herbivorous insects is the necessity to overcome the defense mechanisms of host plants, thereby optimizing their ability to acquire necessary nutrients for survival and reproduction. Glucosinolate sulfatases (GSS) are recognized as pivotal adaptive mechanisms employed by *P. xylostella* to counteract the glucosinolate–myrosinase defense systems present in cruciferous plants [73,74]. *PxGSS1* functions by secreting a desulfurization inhibitor that hinders the formation of harmful compounds derived from the standard plant defensive molecule 4-(methylsulfinyl) butyl glucosinolate (4MSOB-GL). A knockout of *PxGSS1* leads to a reduced capacity for host adaptation, elevated larval mortality rates, and prolonged pupal stage. Conversely, mutation in *PxGSS2* reduces egg hatching rate, increases larval mortality, and prolongs pupal development. Notably, a loss-of-function mutation in *PxGSS3* does not exert a significant effect on host adaptability in *P. xylostella* [49,50,52,75]. Similarly, knockout of the methyltransferase-like 14 gene (*Pxmettl14*), which encodes an RNA transferase gene, markedly impairs host adaptability. This impairment is evidenced by significantly reduced pupal weight, oviposition rate, and egg hatching rate in comparison to the wild type when transitioning from artificial feed to host plants [53]. The upregulation of the glycoside hydrolase family 1 (GH1) member *Px008848* protein reduces the survival of larvae when they feed on host plants. Conversely, the knockout of *Px008848* greatly increases the survival rate of mutant larvae relative to wildtype [50]. Isothiocyanates (ITCs) exhibit both repellent and toxic effects on herbivores. Specialist herbivores, however, utilize volatile ITCs as essential cues for locating the host. The olfactory receptors *Or35* and *Or49* play a significant role in this process. CRISPR/Cas9-mediated mutations in either *PxOr35* or *PxOr49* lead to a decrease in oviposition preference for ITCs, whereas double knockout strains of female adults completely lose their preference for ITCs and show no selective preference between wild-type *Arabidopsis* and ITC-knockout *Arabidopsis* plants [51]. Furthermore, *PxOr16* demonstrates specific sensitivity to heptanal, an epidermal volatile compound released by *Cotesia vestalis*. Knockout of *PxOr16* eliminates the avoidance behavior towards heptanal [56].

Climate change presents novel challenges for temperature-adopting organisms, particularly insects, in relation to global warming. Trehalose acts as the main source of sugar in the hemolymph of insects and has a crucial function in energy metabolism and stress response. In the diamondback moth, knockout of the *PxTret1-like* gene significantly reduces tolerance to extreme temperatures, decreases reproductive capacity, enhances the mortality rate, and hinders development, reproduction, and temperature adaptability [55]. Very-long-chain fatty acids (VLCFAs) are essential for insects to adapt to thermal stress. The *3-hydroxy acyl-CoA dehydratase 2* gene (*Hacd2*) is known as a crucial enzyme in VLCFA synthesis. Knockout of *PxHacd2* results in increased cuticular permeability caused by decreased VLCFA levels in *P. xylostella*. This, in turn, makes them less capable of adapting to dry environmental conditions, leading to significantly reduced survival rates and reproductive capacity [54]. Knockout of peripheral genes *Karyopherin subunit beta 1* (*KPNB1*) and *Toiled-coil domain containing 2* (*GCC2*) weakens the response ability of *P. xylostella* with respect to extreme temperatures while concurrently inhibiting the development and reproduction processes [57].

### 2.6. Insecticide Resistance

The issue of insecticide resistance in *P. xylostella* has significantly escalated, with almost all chemical insecticides showing various levels of resistance. The lack of understanding about the mechanisms underlying this resistance has impeded the advancement of basic strategies to tackle this issue. However, by using CRISPR/Cas9 technology on the *P. xylostella*, targeted mutations have enabled the comprehensive investigation of the mechanisms responsible for its insecticide resistance. Research on the gene mutations associated with resistance has established a foundation for the further elucidation of how pests develop resistance against chemical insecticides. The main target of diamide insecticides is the ryanodine receptor (RyR). Previous studies have identified three specific mutations at positions—i.e., G4946E, I4790K, and I4790M—in RyR that are associated with diamide resistance. Two knock-in strains, PxI4790K-KI and PxI4790M-KI, have been developed using a CRISPR/Cas9-mediated system, with a single-stranded oligonucleotide DNA (ssODNA) of approximately 120 base pairs serving as the knock-in template. Compared to susceptible strains, PxI4790K-KI exhibits significantly increased resistance to cyantraniliprole and chlorantraniliprole, as well as to the diamide flubendiamide. Similarly, PxI4790M-KI demonstrates enhanced resistance to chlorantraniliprole, cyantraniliprole, and flubendiamide, [64,67]. Various kinds of insecticides have the nicotinic acetylcholine receptor (nAChR) as a common target. A CRISPR/Cas9-mediated deletion of *PxnAChRα6* confers significantly increased resistance to Spinosad and spinetoram. However, this mutation does not result in notable changes; it causes only limited increases in resistance to abamectin, imidacloprid, indoxacarb, β-cypermethrin, chlorantraniliprole, and metaflumizone [63]. Cyclopentadiene organochlorine and phenylpyrazole insecticides inhibit neurotransmission in insects by blocking the GABA-gated chloride channel. and it has been confirmed that the A301S mutation in GABA receptors contributes to cyclodiene, organochlorine, and phenylpyrazole pesticide resistance. CRISPR/Cas9-mediated editing of *PxGABARalpha* in an insecticide-susceptible strain of *P. xylostella* does not confer resistance to dieldrin, endosulfan, or fipronil [58]. Knockout studies of two fipronil-sensitive and fipronil-resistant genes (*PxRdl1* and *PxRdl2*) indicate that disruption of *PxRdl1* diminishes the effectiveness of fipronil, while disrupting the fipronil-resistant *PxRdl2* increases the effectiveness of fipronil [76]. The glutamate-gated chloride channel (GluCl) is a primary target for avermectin insecticides. A mutation from valine to isoleucine (V263I) in GluCl has been confirmed to involve varying degrees of resistance for abamectin in the diamondback moth. A CRISPR/Cas9-edited strain containing the homozygous V263I mutation (PxGluClV263I-KI) has been developed, which exhibits significant resistance to avermectin. However, this mutation also results in a notable reduction in reproductive capacity [66].

The fast development of insecticide resistance in pests poses a substantial obstacle to the sustainable utilization of biological pesticides and genetically engineered crops that generate *Bacillus thuringiensis* (*Bt*) toxins. The development of a strong resistance to *Bt* toxins, specifically Cry1Ac, in lepidopteran insects like the diamondback moth has been associated with mutations or the reduced activity of ABC transporter subfamily C genes (ABCC2 and ABCC3). CRISPR/Cas9-mediated knockout strain (PxABCC2-KO) demonstrates a 724-fold increase in resistance, whereas in contrast to the susceptible strain, the PxABCC3-KO strain shows a 413-fold higher resistance to the *Cry1Ac* protoxin. Both knockout strains exhibit a substantial decrease in the binding affinity between the *Cry1Ac* toxin and midgut membrane vesicles compared to the sensitive strain. Furthermore, the loss of these two genes leads to a resistance that is more than 8000 times higher than Cry1Ac and 380 times higher than Cry1Fa. This highlights the fact that ABC transporters serve as redundant toxin receptors in the diamondback moth [51,59,62]. The use of CRISPR/Cas9 technology to simultaneously knockout the *PxAPN1* and *PxAPN3a* results in a remarkable 1425-fold increase in resistance to the *Cry1Ac* toxin. Furthermore, the simultaneous knockout of four genes, namely, *PxABCC2*, *PxABCC3*, *PxAPN1*, and *PxAPN3*, results in an extraordinary increase of over 34000-fold in resistance against *Cry1Ac* toxin [60]. The polycalin protein is known as a possible receptor for *Bt* toxin, and knockout of the *PxPolycalin* gene leads to a decline in the resistance of the diamondback moth to *Cry1Ac* toxin [61]. Methionine aminopeptidase (*MetAP*) plays a crucial role in peptide synthesis, and knockout of *PxMetAP* contributes to increased resistance against *Cry1Ac* toxin in *P. xylostella* [65]. Juvenile hormone binding protein (JHBP) is a pivotal regulatory factor in the signaling pathway of juvenile hormone (JH). Notably, a mutation within *PxJHBP* leads to a substantial increase in sensitivity to *Cry1Ac*, resulting in a shortened lifespan and significantly impaired reproductive capacity [68].

## 3. Application of CRISPR/Cas9 Knock-In and Gene Drive Systems in the Diamondback Moth

**Figure 3:**
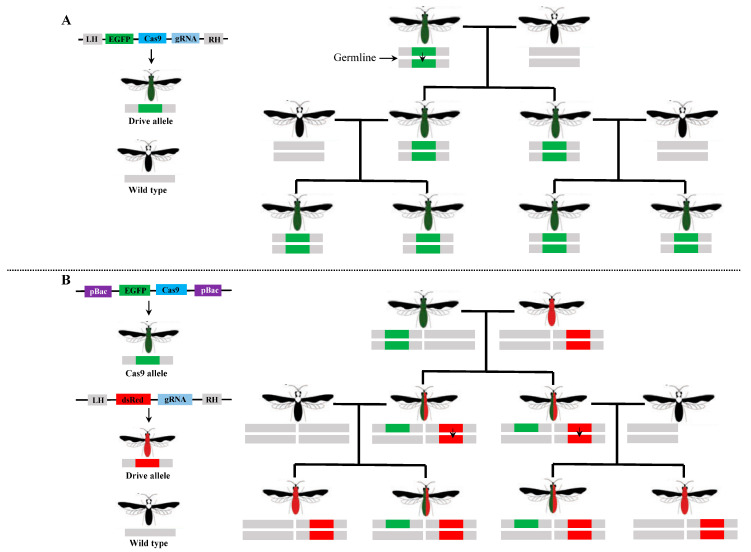
Schematic illustration of gene drive systems. (**A**) depicts the global gene drive where Cas9, gRNA, and a marker gene are inserted into the target locus. Under normal conditions, Cas9 cleaves the target locus in germ cells and converts the normal (wild-type) allele into a gene drive allele that bypasses Mendelian segregation. (**B**) illustrates the split-drive system where Cas9 and one marker gene are inserted into an autonomous chromosome, while gRNA and another marker gene are inserted into the target locus. Two independent transgenic lines are created and crossed. The drive allele is inherited more frequently than the normal Mendelian segregation due to the active presence of Cas9 and gRNA in germline cells. This schematic representation illustrates germline drive conversion, highlighting the process of germline homing.

Gene drive technology holds the potential to rapidly disseminate desirable traits into wild populations, presenting an efficient method for controlling or eradicating target species [77,78,79]. With the continuous advancement of gene-editing techniques, gene drive systems have become a promising approach for pest control applications. Among these approaches, CRISPR/Cas9-based gene drive systems have gained considerable interest because of their simplicity, versatility, and high efficacy [8,80,81]. The successful implementation of CRISPR/Cas9-based gene drives in dipteran insects, specifically in fruit flies and mosquitoes, has opened the possibility of developing a functional gene drive in the diamondback moth. Two types of CRISPR/Cas9-based gene drive systems, namely, the global gene drive system and the split-drive system, have been constructed to modify the population of diamondback moth (Figure 3). In the global gene drive system, all gene drive components, including the *Cas9* gene, gRNA, and marker gene, are integrated into a single construct (Figure 3). Specifically, a global gene drive system in *P. xylostella* has been successfully developed, targeting *PxYellow*, known as a phenotypic marker gene, and gene drive components are precisely integrated into the diamondback moth genome, which are inherited by subsequent generations. The HDR-based efficiency of the gene drive ranges from 6.67% to 12.59%, demonstrating the possibility of developing effective CRISPR/Cas9-based pest control strategies for the diamondback moth. Additionally, it provides a solid foundation for constructing a functional CRISPR/Cas9 gene drive for diamondback moth [69]. In split-drive system, the Cas9 gene along with one marker gene is inserted into autonomous chromosomes using the piggyBac-mediated transposition system, while the gRNA and another marker (drive component) are inserted into the target gene through the CRISPR/Cas9 knock-in system. Two distinct lines, one producing Cas9 and the other producing gRNA, are created and crossed to ensure the inheritance of the drive components in subsequent generations (Figure 3). The Cas9 components are inherited normally, whereas the gRNA components bypass Mendelian segregation. A split-drive gene drive system targeting two phenotypic genes, *PxKmo* and *PxYellow*, has been developed in *P. xylostella*. In this system, different endogenous promoters are tested to drive Cas9 expression, alongside a single gRNA, which results in a high level of cleavage in somatic cells but low cleavage in germline cells and no homing effects. This study lays the groundwork for improving the functional split-drive system, which can be further utilized to develop genetic control methods for *P. xylostella* [70]. In addition to gene drive systems, the CRISPR/Cas9 knock-in method has been utilized to evaluate its ability to cleave DNA in the diamondback moth cell lines and to enable the targeted insertion of marker genes into sex chromosomes for the purpose of separating genders at an early stage. A transgenic diamondback moth cell line expressing Cas9 has been successfully generated using two ubiquitous promoters: Px217-2 and IE1. The Px217-2 promoter exhibits robust gene knockout efficiency compare to the IE1 promoter in diamondback moth cell lines [82]. The use of CRISPR/Cas9 technology to precisely insert marker genes into sex chromosomes allows for the separation of genders, providing a secure and economically efficient approach that may be applied to the mass production of transgenic females or males. Using CRISPR/Cas9 knock-in technology, the DsRed2 gene has been inserted into the W chromosome at a specific genomic locus. However, this insertion severely affects the typical growth and reproduction of the diamondback moth, leading to deformities and infertility, indicating that this particular genomic locus is unsuitable for integrating exogenous sequences. Conversely, the cyan fluorescent protein (CFP), along with the Cas9 gene (Cas9-CFP), has been precisely integrated into another genomic locus on the Z chromosome. The introduction of the Cas9 gene along with CFP marker gene into the Z chromosome enables gender separation through fluorescence labeling. These findings demonstrate the feasibility of integrating exogenous genes into different genomic sites in the diamondback moth [83].

## 4. Challenges and Possible Solutions of CRISPR/Cas9 Gene-Editing in the Diamondback Moth

Although CRISPR/Cas9 has made significant progress in the genome editing of the diamondback moth, it is crucial to acknowledge and address the limitations associated with this technology. These limitations include the possibility of off-target mutation, the delivery of CRISPR/Cas9 components, variations in knockout efficiencies, low germline cleavage, and limited homology-directed repair, which need to be addressed in order to further improve this technology.

The potential for off-target mutations arises from mismatches between sgRNA and its target site, resulting in unintended and nonspecific changes in the genome. The effectiveness of CRISPR/Cas9 depends on the 20 base pair nucleotide sequences of the gRNA and the PAM sequences located close to the target site [19]. Studies have demonstrated that when the gRNA and the off-target sequence have less than six mismatches, this can result in off-target consequences [84,85]. These effects can cause harmful outcomes, such as sequence mutations, deletions, and genome rearrangements, which hinder the application of CRISPR/Cas9 for certain objectives. Although there have been no documented instances of off-target consequences of this system in the diamondback moth, it is hypothesized that such occurrences may still happen and compromise the precision of the CRISPR/Cas9 system. In order to tackle this problem, several approaches may be utilized to improve the precision of the CRISPR/Cas9 system. These include refining the sgRNA sequence, altering the Cas9 gene, and employing alternative Cas variants. Cas variants, such as Cas12a and Cas13, offer unique advantages for genome editing and gene regulation. Cas12a (Cpf1) is an RNA-guided endonuclease that introduces staggered double-stranded breaks, producing cohesive ends that are advantageous for precise gene insertions [86,87]. Unlike Cas9, Cas12a recognizes T-rich PAM sequences, which expand its target range, particularly in AT-rich genomes [88]. Furthermore, it operates using a single crRNA, simplifying the design and delivery of editing components. Cas13, on the other hand, is unique in targeting RNA rather than DNA [89,90]. This makes it particularly useful for transient gene knockdown, studying RNA function, and combating RNA-based pathogens. Its high specificity for RNA reduces the risk of permanent off-target effects, making it a promising tool for applications requiring reversible gene regulation [89]. These variants, with their distinct mechanisms and features, could be helpful in overcoming the off-target challenges of CRISPR/Cas9 while broadening the scope of genetic interventions in *P. xylostella*.

Additionally, delivering CRISPR/Cas9 components into target organisms can be crucial, especially for in vivo applications, and may influence the efficiencies [91]. In the case of *P. xylostella*, the ribonucleoproteins have mainly been utilized as CRISPR/Cas9 components and delivered through embryo microinjections (Table 1). Microinjection to early embryos is a difficult, time-consuming, and laborious procedure. Furthermore, the post-injection survival rate of individuals varies and relies on expertise, which poses a challenge in making this system effective for *P. xylostella* (Table 1 and Table 2). Other delivery methods, including receptor-mediated ovary transduction of cargo (ReMOT) control, have been successfully developed for mosquitoes and *B. mori*. In this method, adult individuals and pupae were injected with Cas9-mediated ribonucleoprotein complexes, resulting in significant germline mutations [92,93]. This method can be optimized for *P. xylostella*, which may help to overcome challenges related to the delivery of components to the diamondback moth. However, its application to *P. xylostella* may present some potential limitations and risks. One major limitation would be the variability in delivery efficiency as the method relies on the compatibility of ligand–receptor interactions, which may not be fully characterized for *P. xylostella*. Additionally, unintended off-target effects could occur if the cargo interacts with non-target tissues or receptors, potentially leading to undesired physiological or developmental changes. Furthermore, the potential for incomplete or inconsistent germline editing may hinder its application in population-level genetic control strategies. Despite these challenges, further research into optimizing ligands and improving delivery specificity could enhance the feasibility of ReMOT control for *P. xylostella* and might offer advantages over traditional germline delivery methods.

The efficiency of gene-editing using the CRISPR/Cas9 system in the diamondback moth depends on the components used for microinjections. For instance, using a ribonucleoprotein complex to knockout the target gene results in a significantly better mutation rate compared to using messenger RNA (mRNA) with synthetic sgRNA and plasmid DNA (Table 2). It is crucial to acknowledge that the editing that occurs in somatic tissues cannot be passed on to the next generation. Only germline mutations can be inherited, making it crucial for the effectiveness of this system in pest management. In *P. xylostella*, high somatic cleavage efficiency, i.e., exceeding 70%, hinders the transmission of mutant alleles to offspring [69,70]. To address this challenge, it is essential for Cas9 to cause high cleavage at the target gene in germ cells rather than in somatic tissues. This can be accomplished by limiting the activity of Cas9 in germline cells, necessitating the use of highly specialized promoters that selectively activate Cas9 in germ tissues.

The main challenge of the CRISPR/Cas9 system is the low effectiveness of the homing-based gene drive and the formation of high-resistance alleles in the diamondback moth. The CRISPR/Cas9 gene drive operates primarily on the principles of HDR, which involve introducing exogenous gene sequences. This leads to the creation of DSBs at the target locus of an organism, followed by repair through HDR. However, in most cases, the cells mainly rely on NHEJ to repair these DSBs, leading to resistance allele formation. The NHEJ repair process causes the formation of alleles that are resistant to gene drive components. This leads to the elimination of the target site for the gRNA and hampers the propagation of gene drive components among populations. In our previous studies, Cas9 efficiently cleaves the target gene; however, the cleaved place is more often repaired through the NHEJ process rather than the HDR process, resulting in the formation of resistance alleles [69,70]. To overcome this challenge, two basic strategies can be employed: inhibiting the NHEJ pathway to promote HDR-mediated repair, and activating the HDR pathway. There are specific genes involved in the NHEJ-mediated DNA repair pathway, such as *DNA-dependent protein kinase* (*DNA-PK*), *Ku80*, *Ku70*, and *ligase 4* (*LIG4*). *DNA-PK* acts as an initial component in recognizing and binding DSBs during NHEJ repair; the Ku70/80 heterodimer plays an important role for initiating the NHEJ pathway by showing a strong affinity for dsDNA ends; and LIG4 is a vital enzyme that controls the NHEJ repair process. Inhibiting *DNA-PK*, *Ku70/80*, and *LIG4* significantly enhances the HDR-mediated repair mechanism in various organisms, including humans, mice, and insects (*B. mori*) [94,95,96,97,98,99,100,101]. Conversely, HDR is a crucial pathway for the precise repair of DSBs. An alternative strategy to shift DNA repair from NHEJ to HDR involves inducing the activation of key components within the HDR machinery [102,103]. The CtIP protein is crucial in the early phases of HDR, and the integration of transgenes by HDR can be improved by combining Cas9 with the N-terminal domain of CtIP [104]. MLN4924 increases CtIP expression levels by inhibiting neddylation, thereby effectively enhancing HDR efficiency [105]. Rad51 plays a crucial role in facilitating DNA strand exchange during HDR. Heterologous expression of Rad51 has been shown to boost HDR efficiency [106]. The G2/M phase of the cell cycle is another critical window during which HDR predominantly occurs. Utilizing small molecules capable of reversibly arresting cells at this stage significantly improves overall HDR efficiency [107,108,109,110].

Beyond improving HDR efficiency, alternative genome-editing strategies offer potential solutions to mitigate resistance allele formation. Prime editing is emerging as a promising approach, enabling precise genetic modification without inducing DSBs [111,112]. Additionally, advanced techniques such as PASTE (programmable addition via site-specific targeting elements) and TwinPE (twin prime editing) facilitate large DNA insertions with high precision, reducing the risk of resistance allele formation [113]. These techniques could be integrated into gene drive systems to enhance their efficiency and stability. Combining HDR enhancement strategies with prime editing and its derivatives could provide a robust framework for improving gene drive efficiency in *P. xylostella*. Future research should explore the feasibility of integrating these advanced genome-editing tools to achieve more stable and effective genetic control strategies while minimizing resistance allele formation. By focusing on these pathways, the challenges related to low HDR efficiency can be addressed in the future, making this system more effective for genetic manipulations in *P. xylostella.*

## 5. Conclusions and Future Perspectives

This review highlights the remarkable progress and applications of CRISPR/Cas9 genome editing in the diamondback moth (*P. xylostella*), a destructive pest responsible for substantial agricultural losses. This technology has facilitated precise genetic modifications, providing critical insights into the molecular mechanisms underlying reproduction, development, insecticide resistance, and host adaptability. Such advancements lay the foundation for innovative, genetic-based pest control strategies that could significantly reduce reliance on chemical insecticides, thereby promoting sustainable agricultural practices for *P. xylostella*. Despite its considerable potential, the widespread application of CRISPR/Cas9 in pest management faces several technical and ecological challenges. Off-target mutations, inefficient delivery methods, variable knockout efficiencies, low germline editing, and low HDR rates remain key obstacles to be addressed. Additionally, concerns regarding unintended ecological consequences, resistance evolution, and the ethical implications of gene drives necessitate stringent risk assessments and regulatory oversight.

Future research should focus on optimizing HDR-mediated repair, enhancing germline transmission, and exploring alternative CRISPR variants such as CRISPR/Cas12 and CRISPR/Cas13 to improve genome editing in *P. xylostella*. Furthermore, integrating CRISPR-based strategies with ecological approaches, such as integrated pest management (IPM) and biological control, will enhance their sustainability and long-term efficacy. By aligning CRISPR/Cas9 applications with ecological and regulatory considerations, this technology holds great promise for revolutionizing pest management. Through these efforts, the full potential of CRISPR/Cas9 in pest management can be achieved, leading to more sustainable and effective solutions for controlling diamondback moth.

## Figures and Tables

**Table 1:** Utilization of CRISPR/Cas9-based system in the diamondback moth.

Applications	Gene	Knockout/ Knock-In	CRISPR/Cas9 Component	Method of Delivery	Gene Function and Disruption Impact	References
Development and reproduction	*PxHomeobox*	Knockout	Cas9 mRNA	Embryo injections	Transcription factor; lethal to embryos	[31]
	*PxVg*	Knockout	Ribonucleoprotein	Embryo injections	Egg maturation; reduced hatching rate	[32]
	*PxVgR*	Knockout	Cas9 mRNA	Embryo injections	Oocyte development; smaller eggs; low fertility	[33]
	*PxSer2*	Knockout	Ribonucleoprotein	Embryo injections	Semen protein; male sterility	[34]
	*PxVMP*	Knockout	Ribonucleoprotein	Embryo injections	Egg structure formation; egg collapse and reduced hatching	[35]
	*PxPiwi*	Knockout	Ribonucleoprotein	Embryo injections	Germ cell development; delayed pupation; failed emergence	[36]
	*PxTAR1*	Knockout	Ribonucleoprotein	Embryo injections	Germ cell development; delayed pupation,;failed emergence	[37]
	*PxOsp*	Knockout	Ribonucleoprotein	Embryo injections	Ovarian development; fewer and irregular eggs	[38]
	*PxDcr-1*	Knockout	Ribonucleoprotein	Embryo injections	RNA processing; increased mortality; reduced reproduction	[39]
	*PxDcr-2*	Knockout	Cas9 mRNA	Embryo injections	RNA processing; reduced RNAi efficiency	[39]
Pigmentation	*PxKMO*	Knockout	Ribonucleoprotein	Embryo injections	Eye pigment gene; yellow/red eye color	[40]
	*PxCardinal*	Knockout	Ribonucleoprotein	Embryo injections	Eye pigment gene; yellow eyes turning red with age	[40]
	*PxEbony*	Knockout	Ribonucleoprotein	Embryo injections	Dopamine metabolism; darker body color	[41]
	*PxYellow*	Knockout	Ribonucleoprotein	Embryo injections	Body pigmentation gene; change in body color	[42]
Sex-determination	*PxDsx*	Knockout	Cas9 mRNA	Embryo injections	Sex differentiation; genital abnormalities	[43]
	*PxPSI*	Knockout	Cas9 mRNA	Embryo injections	Male-specific splicing; genital defects	[44]
	*PxSast1*	Knockout	Ribonucleoprotein	Embryo injections	Sex differentiation; male infertility; egg defects	[45]
Circadian rhythms	*PxCry1*	Knockout	Ribonucleoprotein	Embryo injections	Light-sensitive photoreceptor; altered activity rhythms	[46]
	*PxLW-opsin*	Knockout	Ribonucleoprotein	Embryo injections	Phototaxis; impaired light response	[47]
	*PxCry2*	Knockout	Ribonucleoprotein	Embryo injections	Circadian rhythm; disturb the rhythmic activities	[48]
	*PxPer*	Knockout	Ribonucleoprotein	Embryo injections	Circadian rhythm; activity disruption	[48]
Ecological adaptability	*PxGSS1*	Knockout	Ribonucleoprotein	Embryo injections	Glucosinolate metabolism; reduced host plant adaptation	[49,50]
	*PxGSS2*	Knockout	Ribonucleoprotein	Embryo injections	Glucosinolate metabolism; reduced host plant adaptation	[49,50]
	*PxOr35*	Knockout	Ribonucleoprotein	Embryo injections	Olfactory receptor; oviposition preference reduced	[51]
	*PXOr49*	Knockout	Ribonucleoprotein	Embryo injections	Olfactory receptor; oviposition preference reduced	[51]
	*PxGSS3*	Knockout	Ribonucleoprotein	Embryo injections	Glucosinolate metabolism; host adaptability unchanged	[52]
	*PxMETTL14*	Knockout	Ribonucleoprotein	Embryo injections	RNA methyltransferase; developmental defects	[53]
	*Px008848(PxGH1)*	Knockout	Ribonucleoprotein	Embryo injections	Insect–plant interaction; increased larval survival	[50]
	*PxHacd2*	Knockout	Ribonucleoprotein	Embryo injections	Enzyme for VLCFAs synthesis; survival and fecundity decreased	[54]
	*PxTret1-like*	Knockout	Ribonucleoprotein	Embryo injections	Trehalose transport; reduced temperature resistance	[55]
	*PxOr16*	Knockout	Ribonucleoprotein	Embryo injections	Odor receptor; reduced avoidance behavior to parasitoid	[56]
	*PxGCC2*	Knockout	Ribonucleoprotein	Embryo injections	Peripheral gene for stress; reduced stress resistance	[57]
	*PxKPNB1*	Knockout	Ribonucleoprotein	Embryo injections	Peripheral gene for stress; decreased environmental adaptation	[57]
Insecticide resistance	*PxGABAR-α1 (A282S)*	Knock-in	Cas9 protein crRNA, tracRNA and ssODNA	Embryo injections	GABA-gated chloride channel; not involved in pesticide resistance	[58]
	*PxABCC2*	Knockout	Ribonucleoprotein/Cas9 mRNA	Embryo injections	Bt resistance; reduced Cry1Ac sensitivity	[51,59,60,61,62]
	*PxABCC3*	Knockout	Ribonucleoprotein/Cas9 mRNA	Embryo injections	Bt resistance; reduced Cry1Ac sensitivity	[51,59,60,62]
	*PxnAChRα6*	Knockout	Ribonucleoprotein	Embryo injections	Nicotinic acetylcholine receptor α6; increased pesticide resistance	[63]
	*PxRyR (I4790M)*	Knock-in	Ribonucleoprotein and ssODN	Embryo injections	Ryanodine receptor; increased diamine resistance”	[64]
	*PxAPN1*	Knockout	Ribonucleoprotein	Embryo injections	Aminopeptidases increased Cry1Ac resistance	[60]
	*PxAPN3a*	Knockout	Ribonucleoprotein	Embryo injections	Aminopeptidases increased Cry1Ac resistance	[60]
	*PxMetAP1*	Knockout	Ribonucleoprotein	Embryo injections	Methionine aminopeptidases; impaired peptide processing	[65]
	*PxGluCl (V263I)*	Knock-in	Cas9 proteinss/sgRNA/ssODN	Embryo injections	Glutamate-gated chloride chann; resistance to avermectin	[66]
	*PxPolycalin*	Knockout	Ribonucleoprotein	Embryo injections	Bt toxin receptor; decreased Cry1Ac sensitivity	[61]
	*PxRyR (I4790K)*	Knock-in	Ribonucleoprotein and ssODN	Embryo injections	Ryanodine receptor; chlorantraniliprole resistance	[67]
	*PxJHBP*	Knockout	Ribonucleoprotein	Embryo injections	JH signaling regulator; increased Cry1Ac susceptibility	[68]
Gene drive	*PxYellow*	Knock-in	Ribonucleoprotein and plasmid	Embryo injections	Body pigmentation gene; change in body color	[69,70]
	*PxKmo*	Knock-in	Ribonucleoprotein and plasmid	Embryo injections	Eye pigment gene; yellow/red eye color	[70]

**Table 2:** Efficiency of CRISPR/Cas9 system in the diamondback moth.

Target Gene	No. of Eggs Injected	Survived Individuals	Survival Rate	Mutation Rate	Reference
*PxABCC2*	215	80	37.21%	65.00%	[59]
*PxABCC3*	200	68	34.00%	58.82%	[59]
*PxDsx^C^*	489	151	30.87%	62.91%	[43]
*PxDsx^F^*	431	126	29.23%	34.13%	[43]
*PxDsx^M^*	518	107	20.66%	42.99%	[43]
*PxGSS1*	205	52	25.36%	3.85%	[52]
*PxGSS2*	205	52	25.36%	5.77%	[52]
*PxVgR*	258	122	47.29%	63.11%	[33]
*PxYellow*	676	480	71.01%	56.66%	[42]
*PxSer2*	2000	490	36.5%	1.02%	[34]
*PxKmo*	222	94	42.34	17.02%	[40]
*PxCardinal*	182	55	30.22%	29.09%	[40]
*PxVg*	135	99	73.33%	4.04%	[32]
*PxPSI*	638	324	50.78%	23.15%	[44]
*PxCry1*	263	58	22.05%	20.69%	[46]
*PxMETTL14*	256	30	11.72%	10.00%	[53]
*PxVMP*	52	10	19.23%	22.22%	[35]
*PxTret1-like*	100	62	62%	11.29%	[55]
*PxLW-opsin*	211	62	29.38%	6.45%	[47]
*PxHacd2*	100	56	56.00%	3.57%	[54]
*PxPiwi*	212	98	46.23%	55.10%	[36]
*PxGluCl (V263I)*	300	11	3.67%	9.09%	[66]
*PxTAR1*	154	68	44.16%	13.24%	[37]
*PxOsp*	180	123	68.33%	19.25%	[38]
*PxSast1*	185	68	36.80%	1.47%	[45]
*PxPer*	210	72	34.29%	4.17%	[48]
*PxRyR (I4790K)*	1350	59	4.37%	4.35%	[67]
*PxGCC2*	176	53	30.11%	5.66%	[57]
*PxKPNB1*	154	42	27.27%	9.52%	[57]
